# Investigations into the association between soil-transmitted helminth infections, haemoglobin and child development indices in Manufahi District, Timor-Leste

**DOI:** 10.1186/s13071-017-2084-x

**Published:** 2017-04-19

**Authors:** Suzy J. Campbell, Susana V. Nery, Catherine A. D’Este, Darren J. Gray, James S. McCarthy, Rebecca J. Traub, Ross M. Andrews, Stacey Llewellyn, Andrew J. Vallely, Gail M. Williams, Archie C. A. Clements

**Affiliations:** 1 Research School of Population Health, College of Medicine, Biology and Environment, The Australian National University, 62 Mills Rd, Acton, ACT 2601 Australia; 2 Molecular Parasitology Laboratory, QIMR Berghofer Medical Research Institute, 300 Herston Rd, Brisbane, QLD 4006 Australia; 3 School of Public Health, University of Queensland, Herston Rd, Brisbane, QLD 4006 Australia; 4 Clinical Tropical Medicine Laboratory, QIMR Berghofer Medical Research Institute, 300 Herston Rd, Brisbane, QLD 4006 Australia; 5 Faculty of Veterinary and Agricultural Science, The University of Melbourne, Parkville, VIC 3010 Australia; 6 Menzies School of Health Research, Charles Darwin University, Ellengowan Dr, Casuarina, NT 0810 Australia; 7 Kirby Institute, University of New South Wales, Wallace Wurth Building, High St, Kensington, NSW 2052 Australia

**Keywords:** Soil-transmitted helminths, *Necator americanus*, *Ascaris*, Morbidity, Anaemia, Stunting, Wasting, PCR

## Abstract

**Background:**

Timor-Leste has a high prevalence of soil-transmitted helminth (STH) infections. High proportions of the population have been reported as being anaemic, and extremely high proportions of children as stunted or wasted. There have been no published analyses of the contributions of STH to these morbidity outcomes in Timor-Leste.

**Methods:**

Using baseline cross-sectional data from 24 communities (18 communities enrolled in a cluster randomised controlled trial, and identically-collected data from six additional communities), analyses of the association between STH infections and community haemoglobin and child development indices were undertaken. Stool samples were assessed for STH using qPCR and participant haemoglobin, heights and weights were measured. Questionnaires were administered to collect demographic and socioeconomic data. Intensity of infection was categorised using correlational analysis between qPCR quantification cycle values and eggs per gram of faeces equivalents, with algorithms generated from seeding experiments. Mixed-effects logistic and multinomial regression were used to assess the association between STH infection intensity classes and anaemia, and child stunting, wasting and underweight.

**Results:**

Very high stunting (60%), underweight (60%), and wasting (20%) in children, but low anaemia prevalence (15%), were found in the study communities. STH were not significantly associated with morbidity outcomes. Male children and those in the poorest socioeconomic quintile were significantly more likely to be moderately and severely stunted. Male children were significantly more likely than female children to be severely underweight. Increasing age was also a risk factor for being underweight. Few risk factors emerged for wasting in these analyses.

**Conclusions:**

According to World Health Organization international reference standards, levels of child morbidity in this population constitute a public health emergency, although the international reference standards need to be critically evaluated for their applicability in Timor-Leste. Strategies to improve child development and morbidity outcomes, for example *via* nutrition and iron supplementation programmes, are recommended for these communities. Despite the apparent lack of an association from STH in driving anaemia, stunting, wasting and underweight, high endemicity suggests a need for STH control strategies.

**Trial registration:**

Australian and New Zealand Clinical Trials Registry ACTRN12614000680662; retrospectively registered.

**Electronic supplementary material:**

The online version of this article (doi:10.1186/s13071-017-2084-x) contains supplementary material, which is available to authorized users.

## Background

Southeast Asia harbours one-third of the world’s soil-transmitted helminths (STH) [1], and Timor-Leste is one of the poorest countries in the region [2]. Two recent cross-sectional studies identified moderate school-based (29%) [3] and high community-based (69%) [4] STH prevalence in the Manufahi District of Timor-Leste, using different diagnostic techniques. Specifically, in this area, prevalence of *Necator americanus* was 60%, *Ascaris* spp. 24%, *Ancylostoma* spp. 4.7%, and *Trichuris trichiura* 0.33%, with *Giardia duodenalis* the most common protozoan identified (13%) [4]. Inadequate water and sanitation infrastructure and hygiene behaviours in this area likely contribute to high STH endemicity [4], which in turn could contribute significantly to morbidity.

STH have previously been associated with anaemia, stunting and wasting [5–9]. The mechanism whereby hookworms contribute to reduced haemoglobin and more indirectly to poor growth and development outcomes is *via* blood loss and inflammation, with heavily-infected people at greatest risk of morbidity [10]. *Ascaris lumbricoides* is not considered a contributor to blood loss (reviewed in [11]) and *T. trichiura* contributes to blood loss in heavy infection. Whilst STH have been shown to be associated with stunting and wasting [12, 13] the causal relationship is not clear.

Despite inability to establish causality with observational analyses, investigating the relationship between STH infection and haemoglobin concentration, and child anthropometric indices, is of considerable importance in Timor-Leste. Extremely high proportions of Timorese under 5 years of age are reported as stunted (50%) and wasted (11%) [14], yet knowledge of the contribution of STH to this is very limited, with no prior investigations identified. A cross-sectional survey in 2008 found 22% of children aged 24–59 months of age were anaemic [15]. Additionally, a demographic health survey in 2009–2010 found 38% of Timorese children aged 6–59 months, and 21% of Timorese women aged 15–49 years were anaemic [16]. Since this time, risk factors for anaemia in women of reproductive age have been investigated [17]. However, limited data on STH have prevented STH contributions from being assessed.

Using quantitative polymerase chain reaction (qPCR) for STH diagnosis and intensity of infection assessment [18], we aim to (i) determine classes of STH infection intensity from PCR-derived data, and (ii) provide the first analysis of morbidity associated with STH infections in Manufahi District, Timor-Leste. We used an algorithm to correlate quantification cycle (C_q_) values from qPCR to eggs per gram of faeces (epg) equivalents, determined by seeding experiments [18]. The association between intensity of *N. americanus* and *Ascaris* spp. (as exposures) and mean haemoglobin concentrations and anaemia diagnosis (as outcomes) were then investigated for all community members, and associations with stunting, underweight and low BMI-for-age (as a measure of wasting) as outcomes in children aged one to 18 years were also investigated.

## Methods

### Study setting, design and collection of data

This study was conducted as one of a series of baseline analyses for the “WASH for Worms” RCT, which aims to determine the extent of a reduction in burden of STH by integrating mass chemotherapy and community-based water, sanitation and hygiene (WASH) programmes [19]. Rural communities in Manufahi District were selected for the study according to RCT-related inclusion and exclusion criteria (including being identified by the Timorese government as high-priority communities for WASH interventions) [19]. The RCT commenced in May 2012, with baseline surveys conducted in 18 communities until October 2013. Identically-collected data from six communities in Manufahi District were added; these communities were enrolled at the same time as the RCT communities but were not randomly allocated to each trial arm. Full details of study area and design [19], questionnaires and parasitological diagnostic approaches [4, 18] are provided elsewhere. Briefly, Manufahi District is comprised of rural Timorese villages with subsistence-based livelihoods. Community consultations and consent elicitation were conducted before questionnaire administration. Children aged less than 12 months and pregnant women in the first trimester of pregnancy were excluded because they could not receive albendazole. Questionnaires were used to record details of water, sanitation and hygiene (WASH) practices, household and individual socioeconomic characteristics [4].

### Measurement of anaemia status and anthropometry

Anaemia status was measured for all ages with haemoglobin concentration assessed by finger-prick blood test using a portable haemoglobinometer (HemoCue, Ängelholm, Sweden). Haemoglobin concentrations can be used to assess anaemia (being inadequate intakes and reserves of host iron and protein [20]). Data on haemoglobin were linked to household GPS coordinates and adjusted by -2 grams per litre for elevation of 1000 m above sea level in accordance with World Health Organization (WHO) recommendations [21]; data from four communities, and part of a fifth community, were adjusted in this way; no communities had an elevation above 1500 m. Haemoglobin was initially classified based on WHO definitions of anaemia severity (Table 1 [21]); however due to small numbers was re-categorised as a binary variable (anaemic/non-anaemic); which was used as the primary outcome.

**Table 1:** Definitions of anaemia used in this study, measured as grams per litre (source [23])

	No anaemia	Mild anaemia	Moderate anaemia	Severe anaemia
Children < 5 years	≥ 110	100–109	70–99	< 70
Children 5–11 years	≥ 115	110–114	80–109	< 80
Children 12–14 years	≥ 120	110–119	80–109	< 80
Non-pregnant women (≥ 15 years)	≥ 120	110–119	80–109	< 80
Pregnant women	≥ 110	100–109	70–99	< 70
Men (≥ 15 years)	≥ 130	110–129	80–109	< 80

For children aged two to < 18 years, weight was measured to the nearest 0.1 kg using electronic scales (CAMRY, ED-301), and height was measured to the nearest 0.1 cm using a portable stadiometer (Wedderburn, WSHRP). Children aged 1 to 2 years had length measured supine with a measuring mat (Wedderburn, SE210), and weight measured by taring (i.e. with the child held by an adult, and the adult’s weight subsequently deducted).

Height-for-age (HAZ) and BMI-for-age (BMIZ, i.e. weight over height^2^-for-age) *z*-scores were calculated for children aged 1– < 18 years. Weight-for-age (WAZ) *z*-scores were calculated for children aged 1– < 10 years, standardised to the international 2006 reference population using the software WHO Anthro and Anthroplus, for children up to five and aged five and over, respectively [22, 23]. These scores are expressed as differences from the reference median and are calculated based on sex and date of birth of each individual. Age in days was used for z-score calculations. Children of uncertain birthdate were assigned a mid-year birthdate (15th June) and records followed up with parents subsequently; 1038 children’s records (95.4%) had completed birthdates. WAZ is only calculated up to 10 years of age, because it is considered inadequate for monitoring growth beyond this age [24]; BMIZ complements HAZ in the assessment of thinness (low BMIZ) [24], and was used instead of weight-for-height (which is calculated for under-fives only) to assess wasting. Each of these continuous outcomes was categorised, with individuals classified as moderately stunted, underweight or wasted if HAZ, WAZ and BMIZ respectively were more than two standard deviations below the reference median, and severely stunted, underweight or wasted, respectively, if the *z*-scores were more than three standard deviations below the reference median [25].

### Assessment of STH infection

Single stool samples were collected, preserved in 5% potassium dichromate at room temperature, transported to QIMR Berghofer Medical Research Institute, Brisbane, Australia, and tested by multiplex qPCR for presence and intensity of STH and protozoal infection using a method previously described [18]. Prevalence of all STH were assessed in this way. Based on highest prevalences, *N. americanus* and *Ascaris* spp. (reported at genus level) were analysed for intensity of infection. For purposes of this analysis, qPCR quantification cycle (C_q_) values, representing the amplification cycle where the signal exceeded background, was interpolated as a measure of the parasite DNA load in the stool sample [26], using a validated internal control. C_q_-values were expressed on a log_10_ scale of the linear equation with fluorescence (i.e. log(*b*_0_ + *b*_1_ *x*)), where the slope (*b*_1_) and the y-intercept (*b*_0_) are provided from the PCR output and *x* is the C_q_-value. Lower values therefore denote heavier intensity infection. In these assays a C_q_-value of 31 for *Ascaris*, and a C_q_-value of 35 for *N. americanus*, were set as the limits for detection of infection [18]. All assays showed C_q_-values for the internal control within the expected range.

### Data analysis

Data were analysed in STATA 14.0 (Stata Corporation, College Station, Texas). A wealth quintile was constructed using principal components analysis of variables assessing ownership of household assets (including animals, transport and appliances), house floor type, reported income and presence of electricity as reported previously [4], according to established methods [27].

For faecal specimens, two runs were taken for each PCR assay. The arithmetic mean of the two (untransformed) C_q_-values was taken to create a single measure per person. For ease of interpretation and for comparison with other studies, untransformed C_q_-values were categorised in these analyses. Receiver-operating characteristic curves (ROCs) were used to assign initial cut-points for C_q_-values, using a generated morbidity score (see Additional file 1). However, a very weak relationship between intensity of infection for either *N. americanus* or *Ascaris* and the morbidity score was observed (see below). This led to extremely poor predictive capacity using ROCs, so this technique was not ultimately used. Full detail of this is reported in Additional file 1, as statistical assignment of categories to infection intensities represent an important contribution that may be useful in assigning qPCR data to intensity of infection categories elsewhere.

An algorithm to assign intensity of infection based on approximations of epg was used for this analysis, with intensity classes being based on those endorsed by the WHO to represent high, moderate and low-intensity infections [28] (see Additional file 1 for detail). This algorithm was generated from seeding experiments as previously described [18] and was based on the linear relationship between the log_10_ of epg and C_q_-value [18]. Because WHO endorsed categorisations of epg intensity are based on the Kato-Katz diagnostic technique [28], a recovery factor of 0.2 was applied to current epg classes of infection intensity (based on a 20% recovery rate determined for faecal flotation of *Ascaris* eggs (R. Traub, unpublished data). This recovery factor was used in the absence of a recovery factor being known for Kato-Katz and the known poor accuracy of this technique in diagnosing STH infections [29]) (Table 2).

**Table 2:** *Ascaris* spp. and *Necator americanus* intensity of infection quantification cycle (C_q_) cut-points between heavy and moderate morbidity

Soil-transmitted helminth	Eggs per gram of faeces (epg) class^a^	EPG class with recovery factor applied^b^	Corresponding C_q_-value^c^
*N. americanus*	≥ 4000	20,000	24.6
*Ascaris* spp.	≥ 50,000	250,000	15.4

*Abbreviations*: C_q_, quantification cycle; epg, eggs per gram of faeces

^a^Eggs per gram of faeces intensity classes follow WHO definitions [28]

^b^Recovery factor of 0.2 applied to epg intensity class based on recovery factor determined from faecal flotation (R. Traub, unpublished data)

^c^DNA intensity from exponentiated C_q_-values

Using classes of infection intensity for epg based on international standards [28], the C_q_ cut-point that correlated with heavy-intensity infection of (≥ 4000*5 = 20,000) epg was selected for *N. americanus*, and the C_q_ cut-point that correlated with heavy-intensity infection of (≥ 50,000*5 = 250,000) epg was selected for *Ascaris*. The final intensity of infection variables for both STH were therefore categorised according to heavy-intensity, moderate- to low-intensity (hereafter called “moderate-intensity”), and no infection, whereby moderate-intensity infection was all C_q_ between the heavy-infection cut-point and the detectable C_q_ limits of 31 for *Ascaris*, and 35 for *N. americanus*. Sensitivity analyses were then undertaken, comparing these cut-points to a model that applied a cut-point of 10% heavy-intensity infection (based on the percentage of endemic populations deemed by the WHO as likely to suffer morbidity from heavy infections [28]), a model of 15% heavy-intensity infection, a model based on quintiles of infection intensity, and also comparing results from applying cut-points that have been reported elsewhere [30–32] to these data. The intensity cut-points selected using the WHO endorsed thresholds as estimated by the algorithm performed comparably to these other cut-points on sensitivity analyses based on parameter estimates and Akaike’s Information Criteria, and thus were used in all further analyses.

Chi-square tests were conducted to compare prevalence of morbidity by age, sex and socioeconomic quintile; these variables were retained as core variables in all multivariable models. The associations between intensity of infection and morbidity by age group and sex were also explored. Mixed-effects logistic regression (for binary-coded anaemia as outcome) and mixed-effects multinomial regression within a generalised structural equation model framework (separately for outcomes of stunting, wasting and underweight) were undertaken to account for correlation among outcomes at the household and village level.

Initially univariable analyses were undertaken with each STH species, *G. duodenalis*, age group (categorical), sex as a binary variable, and socioeconomic quintile (categorical) included as explanatory variables for each of the morbidity outcomes. Although there was a high prevalence of *N. americanus* in this population [4], there was no multicollinearity with other STH species. Variables with *P <* 0.2 from univariable analyses were added stepwise into a base model which included age group, sex, socioeconomic quintile, the categorical intensity of infection explanatory variables for *N. americanus* and *Ascaris* (described above), and binary *Ancylostoma* infection, until the most parsimonious adjusted model for each outcome was achieved. Variables were retained in final models if *P* < 0.1 on the Wald test.

For models of stunting, underweight and wasting, anaemia was additionally included as a core variable because of its importance as a potential confounder. There were insufficient observations to investigate anaemia risk factors in children under five or within other childhood age groups. Anaemia was therefore modelled separately for children (aged 1– < 18 years) and adults, given the difference in prevalence and potential risk factors for these two groups. There were additionally insufficient observations to investigate stunting, wasting or underweight by age groups in regression analyses. Prevalence of both STH and morbidity vary with age and sex, and both the literature [33] and our earlier analyses [4] indicated the potential for moderation of the STH-morbidity relationship by age and sex. Given this, and observed differences in relationships between classes of *N. americanus* infection intensity and stunting across age and sex (identified in cross-tabulations), interactions between sex and *N. americanus* intensity of infection, and age group and *N. americanus* intensity of infection, were investigated. Interactions were investigated by generating models with and without the interaction term and comparing these using the likelihood ratio test, with a requirement for *P* < 0.1 for likelihood ratio tests, for interaction inclusion in the model. Using these criteria, no interactions were required in adjusted models for anaemia. A sex by *N. americanus* intensity of infection interaction was retained in the adjusted child stunting model, and an age group by *N. americanus* intensity of infection interaction was retained in the adjusted child underweight model. For the multinomial child anthropometry models, sensitivity analyses using binary-coded prevalence of outcome (of main effects only, i.e. no interaction terms) were undertaken to investigate the impact of increasing power. Additionally, post-hoc calculations were performed to determine the power to detect effects within each outcome, adjusting for correlations within households and villages. These calculations indicated 80% power, with a 5% significance level, to detect odds ratios of 3.3 or more for anaemia outcomes, relative risks of 1.4 to 1.7 for stunting and underweight outcomes (depending on level of severity), and (reflecting lower numbers) relative risks of 2.1 to 9.5 for wasting as an outcome (according to level of severity).

## Results

### Prevalence of morbidity

**Fig. 1:**
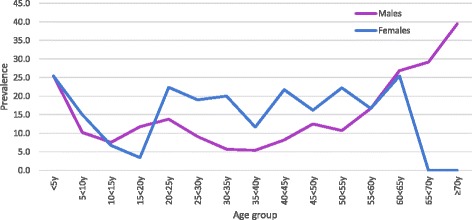
Anaemia distribution by sex and age group (*n* = 2000)

Respondents from communities who provided both a stool and finger-prick blood sample were included in analyses of haemoglobin (2038 participants). Only 15% of the population suffered from anaemia, with the majority of these (11%) being only mildly anaemic, and only three children being severely anaemic (Tables 3 and 4). Anaemia was most prevalent in younger ages, and generally decreased with increasing age (*P* < 0.0001) (Fig. 1). The observed zero prevalence of anaemia in females aged 65 years and over is of interest, although participant numbers in older age groups were generally low. Adult women of reproductive age (aged 18– < 45 years) had higher prevalence of anaemia than men of the same age (18.0 *vs* 7.9%, *P* < 0.0001). The overall prevalence of *N. americanus* and *Ascaris* were 61, and 24%, respectively [4]. The prevalence of *Ascaris* was highest amongst children of preschool age, whereas *N. americanus* was most prevalent in adults (Table 3) [4].

**Table 3:** Baseline characteristics of study participants (*N* = 2038)

Baseline community characteristics	All ages (*N* = 2038)	Children; aged 1– < 18 years (*N* = 1018)	Adults; aged ≥ 18 years (*N* = 1020)
	*n* (%)	*n* (%)	*n* (%)
Mean haemoglobin (g/L, (SD))	131 (16)^a^	126 (13)^a^	136 (16)^a^
Non-anaemic	1 731 (85)	872 (86)	859 (84)
Mildly anaemic	222 (11)	95 (9.3)	127 (13)
Moderately/severely anaemic^b^	86 (4.2)	51 (5.0)	33 (3.3)
*Ascaris* spp. prevalence	501 (25)	302 (30)	199 (20)
*N. americanus* prevalence	1 238 (61)	522 (51)	716 (70)
*Ancylostoma* spp. prevalence	99 (4.9)	44 (4.3)	55 (5.4)
*G. duodenalis* prevalence	261 (13)	203 (20)	58 (5.7)

*Abbreviations*: *g/l* grams per litre; *SD* standard deviation

^a^Mean and standard deviation presented instead of *n* and %

^b^Moderate and severe anaemia categories combined to maintain participant confidentiality

**Table 4:** Anthropometric characteristics of study participants (*N* = 2038)

*Z*-score characteristics				
Height-for-age (HAZ)^a^	All children 1– < 18 years (*N* = 983) *n* (%)	1– < 5 years (*N* = 267) *n* (%)	5– < 10 years (*N* = 365) *n* (%)	≥ 10 years (*N* = 351) *n* (%)
Mean HAZ (SD)^a^	-2.25 (1.22)^a^			
Not stunted	391 (40)	105 (39)	164 (45)	122 (35)
Moderately stunted	347 (35)	89 (33)	132 (36)	126 (36)
Severely stunted	245 (25)	73 (27)	69 (19)	103 (29)
Weight-for-age (WAZ)^b^	All children 1– < 10 years (*N* = 639) n (%)	1– < 5 years (*N* = 268) n (%)	5– < 10 years (*N* = 371) n (%)	
Mean WAZ (SD)^b^	-2.19 (1.03)^a^			
Not underweight	257 (40)	124 (46)	133 (36)	
Moderately underweight	253 (40)	108 (40)	145 (39)	
Severely underweight	129 (20)	36 (13)	93 (25)	
BMI-for-age (BMIZ)^a^	All children 1– < 18 years (*N* = 985) *n* (%)	1– < 5 years (*N* = 266) *n* (%)	5– < 10 years (*N* = 369) *n* (%)	≥ 10 years (*N* = 350) *n* (%)
Mean BMIZ (SD)^a^	-1.19 (1.03)^a^			
Not wasted	796 (81)	242 (91)	308 (84)	246 (70)
Moderately wasted	145 (15)	20 (7.5)	47 (13)	78 (22)
Severely wasted	44 (4.5)	4 (1.5)	14 (3.8)	26 (7.4)

*Abbreviations*: *HAZ* height-for-age, *WAZ* weight-for-age, *BMIZ* BMI-for-age, *SD* standard deviation

^a^HAZ (stunting) and BMI (wasting) calculated for individuals 12 months to < 18 years of age

^b^WAZ (as indicator of underweight) only calculated for individuals 12 months to 10 years of age

Children 1– < 18 years old who provided stool and had height and/or weight measured were included in analyses of *z*-scores (Table 4). Extremely high levels of stunting (60%), underweight (60%), and wasting (20%) were found, with 25% being severely stunted, 20% severely underweight, and 4.5% severely wasted (Table 4). This morbidity is reflected in the mean *z*-scores for each measure, all of which are well below zero. Stunting, underweight and wasting varied by age group with, generally, greater proportions of older children (≥ 10 years) experiencing severe morbidity compared to younger age groups. The prevalence of stunting was significantly higher among poorer households compared to wealthier households (*P* < 0.0001) and among males compared to females (*P* < 0.0001), but the overall association between stunting and age was non-significant (*P* = 0.117). Exploratory analyses indicated some unexpected, but not statistically significant, trends. A greater proportion of uninfected males were severely stunted than *N. americanus*-infected males (Table 5). This trend did not exist for females, who instead showed greater proportions of uninfected having normal (i.e. non-stunted) growth. Similarly, children aged 1 to 5 years with *N. americanus* infection generally had lower prevalence of severe stunting; a trend that reversed in the oldest age group (where a lower proportion of severe stunting was seen in uninfected children (Table 5). These complex and varying underlying relationships confirmed our decision to investigate interaction terms using regression models.

**Table 5:** Baseline characteristics of children by category of stunting (*N* = 592)

Baseline community characteristics	Not stunted *n* (%)	Moderately stunted *n* (%)	Severely stunted *n* (%)
Males			
High *N. americanus* infection intensity	92 (36)	96 (37)	70 (27)
Moderate *N. americanus* infection intensity	10 (31)	10 (31)	12 (38)
No *N. americanus* infection	57 (27)	80 (38)	76 (36)
Total	159 (32)	186 (37)	158 (31)
Females			
High *N. americanus* infection intensity	80 (48)	54 (32)	35 (21)
Moderate *N. americanus* infection intensity	16 (39)	17 (41)	8 (20)
No *N. americanus* infection	136 (50)	90 (33)	44 (16)
Total	232 (48)	161 (34)	87 (18)
Age group 1 to 5 years			
High *N. americanus* infection intensity	38 (48)	27 (34)	14 (18)
Moderate *N. americanus* infection intensity	12 (43)	11 (39)	5 (18)
No *N. americanus* infection	89 (38)	79 (34)	65 (28)
Total	139 (41)	117 (34)	84 (25)
Age group 6 to 11 years			
High *N. americanus* infection intensity	94 (43)	79 (36)	47 (21)
Moderate *N. americanus* infection intensity	10 (35)	11 (38)	8 (28)
No *N. americanus* infection	78 (43)	62 (34)	41 (23)
Total	182 (42)	152 (35)	96 (22)
Age group 12 to 17 years			
High *N. americanus* infection intensity	40 (31)	44 (34)	44 (34)
Moderate *N. americanus* infection intensity	4 (25)	5 (31)	7 (44)
No *N. americanus* infection	26 (38)	29 (42)	14 (20)
Total	70 (33)	78 (37)	65 (31)

Prevalence and severity of being underweight was moderately higher for males (*P* = 0.004), and generally increased by age (*P* = 0.002), but not socioeconomic quintile (*P* = 0.088). Prevalence and severity of wasting increased by age (*P* < 0.0001), but did not differ by sex (*P* = 0.651) or socioeconomic quintile (*P* = 0.666).

### Assignment of DNA intensity cut-points

From PCR output, there were no C_q_-values above 35, indicating good reproducibility of the assays. There were very weak relationships between STH infection and all morbidity outcomes, which hampered statistical assignment of cut-points using ROC curves. Table 2 shows the final selected cut-points. Using our cut-points, 1155 (52%) of study subjects were categorised as having heavy-intensity *N. americanus* infection, and 191 (8.6%) moderate-intensity infection (Table 6). For *Ascaris*, 220 (9.9%) people had heavy-intensity infection, and 318 (14%) people moderate-intensity infection. Amongst infected people, *N. americanus* mean infection intensity was C_q_ 21.5 (95% confidence interval, CI: 21.3–21.8), and for *Ascaris* 19.5 (95% CI: 19.2–19.9) (Table 6). Intensity of infection changed over age, with most heavy-intensity *Ascaris* infection occurring in young children. Heavy-intensity *N. americanus* infections were more evenly distributed across age groups, including older age groups.

**Table 6:** *Necator americanus* and *Ascaris* spp. intensity of infection profile. Numbers indicate all those who provided a stool sample, and therefore are not consistent with the numbers used in separate morbidity analyses

STH	Infection intensity profile *n* (%)			Mean C_q_ (95% CI)
	Heavy intensity	Moderate to low intensity	No infection	
*N. americanus*	1155 (52.0)	191 (8.6)	873 (39.0)	21.5 (21.3–21.8)
*Ascaris* spp.	220 (9.9)	310 (14.0)	1689 (76.0)	19.5 (19.2–19.9)

### Factors associated with anaemia

Neither *Ascaris* nor *N. americanus* infection intensity were significantly associated with anaemia in the multivariable models, although *Ascaris* moderate-intensity infection was marginally non-significant as a risk factor for adults (Table 7). Heavy *N. americanus* infection in children had a protective association with anaemia. However, this was not a significant factor in adjusted models, potentially indicating the confounding effect of other model factors. Increasing age was a highly significant, strongly protective factor in univariable and adjusted models for children. There was no sex difference in odds of anaemia in children. Children in the poorest socioeconomic quintile had twice the odds of anaemia relative to those in the wealthiest quintile. For adults, age was non-significant, and neither sex nor socioeconomic status were associated with anaemia, with no evidence of an overall trend in odds with decreasing socioeconomic quintile.

**Table 7:** Odds ratios for anaemia, by *Necator americanus* and *Ascaris* spp. infection intensity, Manufahi District, Timor-Leste. Logistic regression was used for investigating factors associated with anaemia. The outcome variable is binomial anaemic/non-anaemic and associations therefore presented as odds ratios

	Children; aged 1 < 18 years (*N* = 1018)					Adults; aged ≥ 18 years (*N* = 1020)				
	Univariable		Multivariable			Univariable		Multivariable		
Parameter	OR	95% CI	AOR	95% CI	*P*	OR	95% CI	AOR	95% CI	*P*
*Ascaris* heavy-intensity	0.93	0.49–1.8	0.91	0.46–1.8	0.8820	1.7	0.84–3.3	1.8	0.86–3.6	0.1165
*Ascaris* moderate-intensity	0.73	0.39–1.4	0.85	0.44–1.6		1.7**	1.04–2.9	1.6	0.93–2.7	
*N. americanus* heavy-intensity	0.52***	0.33–0.83	0.69	0.41–1.1	0.1606	0.92	0.62–1.4	0.86	0.56–1.3	0.7280
*N. americanus* moderate-intensity	0.42^a^	0.18–1.0	0.50^a^	0.21–1.2		0.99	0.52–1.9	1.0	0.52–1.9	
*Ancylostoma* infection	1.5^a^	0.58–3.9	1.5^a^	0.58–4.1	0.3940	0.93^a^	0.42–2.1	0.68^a^	0.27–1.7	0.4130
*G. duodenalis* infection	0.92	0.56–1.5				0.42^a^	0.16–1.1			
Male sex	1.1	0.70–1.6	0.97	0.64–1.5	0.8940	0.93	0.66–1.3	0.98	0.68–1.4	0.9180
Age group 6 to 11 years	0.35***	0.22–0.54	0.39	0.24–0.61	< 0.0001					
Age group 12 to 17 years	0.20***	0.11–0.38	0.19	0.09–0.38	< 0.0001					
Age group 65+ years						1.2	0.74–1.9	1.2	0.76–2.0	0.3920
Socioeconomic quintile 4	1.0	0.49–2.0	0.93	0.46–1.9	0.0098	0.58	0.31–1.1	0.58	0.31–1.1	0.3718
Socioeconomic quintile 3	0.52	0.23–1.2	0.49	0.22–1.1		0.85	0.48–1.5	0.83	0.46–1.5	
Socioeconomic quintile 2	1.2	0.60–2.4	1.1	0.55–2.2		1.1	0.65–2.0	1.1	0.62–1.9	
Socioeconomic quintile 1 (poorest)	2.1**	1.0–4.1	2.1	1.0–4.3		0.97	0.54–1.7	0.91	0.50–1.7	

*Notes*: *Ascaris* (categorical infection intensity), *N. americanus* (categorical infection intensity), *Ancylostoma* infection prevalence (binary), age group, sex and socioeconomic quintile were included in all multivariable regression models as exposure outcomes and covariates. Reference categories: no *Ascaris* infection, no *N. americanus* infection, no *Ancylostoma* infection, no *Giardia* infection, age group 1–5 years (for child model), age group 18–64 years (for adult model), male sex, socioeconomic quintile 5 (wealthiest)

*Abbreviations*: *OR* odds ratio, *AOR* adjusted odds ratio, *CI* confidence interval; *P*, Wald test *P*-value

**P < 0.05, ***P < 0.01 in univariable analysis

^a^Less than 10 observations in a subgroup; result should be interpreted cautiously

*Ascaris* and *N. americanus* intensity infections were defined according to following cut-points: *Ascaris*: heavy-intensity C_q_ ≤ 15.4, moderate-intensity C_q_ > 15.4 < 31, no infection C_q_ ≥ 31; *N. americanus*: heavy-intensity C_q_ ≤ 24.6, moderate-intensity C_q_ > 24.6 < 35, no infection C_q_ ≥ 35

### Factors associated with stunting

No level of either *N. americanus* or *Ascaris* infection intensity was associated with stunting of any severity in this population (Table 8). However, whilst not statistically significant, heavy-intensity *Ascaris* infection was associated with higher relative risks for both moderate and severe stunting; these relative risks were of reasonable size (moderate stunting adjusted relative risk (ARR) 1.6, 95% CI: 0.87–2.9; severe stunting ARR 2.0, 95% CI: 0.87–4.4). Sensitivity analysis of stunting prevalence showed that heavy-intensity *Ascaris* infection was marginally non-significantly associated with greater odds of stunting compared to uninfected children (adjusted odds ratio (AOR) 1.8, 95% CI: 0.98–3.4, *P* = 0.057; results not shown). In the multinomial stunting model (Table 8), *Ancylostoma* infection was associated with moderate and severe stunting. However, the effect was in the opposite direction to anticipated: children with *Ancylostoma* infection were significantly less likely to have moderate or severe stunting than uninfected children.

**Table 8:** Relative risk ratios for stunting, Manufahi District, Timor-Leste

	Moderate stunting (*n* = 347)					Severe stunting (*n* = 245)				
Parameter	RR	95% CI	ARR	95% CI	*P*	RR	95% CI	ARR	95% CI	*P*
*Ascaris* heavy-intensity	1.5	0.83–2.7	1.6	0.87–2.9	0.3007	1.8	0.79–4.0	2.0	0.87–4.4	0.2002
*Ascaris* moderate-intensity	1.1	0.65–1.8	1.0	0.61–1.7		1.0	0.50–2.0	0.93	0.46–1.9	
*N. americanus* heavy-intensity^a^	0.82	0.56–1.2	0.82	0.47–1.4	0.6140	0.73	0.43–1.2	0.91	0.41–2.0	0.8647
*N. americanus* moderate-intensity^a^	0.86	0.42–1.7	1.3	0.52–3.1		0.67	0.27–1.7	0.70	0.18–2.7	
Male sex^b^	2.0***	1.4–2.8	2.8	1.7–4.7	< 0.0001	3.7***	2.3–6.0	6.8	3.4–13.7	< 0.0001
*N. americanus* heavy-intensity infection in males^c^			0.65	0.31–1.4	0.2540			0.31	0.11–0.85	0.0230
*N. americanus* moderate-intensity infection in males^c^			0.43	0.10–1.8	0.2440			0.82	0.13–5.3	0.8340
*Ancylostoma* infection	0.26***^d^	0.11–0.65	0.27^d^	0.11–0.68	0.0050	0.33**^d^	0.11–0.99	0.37^d^	0.12–1.1	0.0780
*Giardia* infection	**1.5**	0.96–2.3				1.1	0.61–2.0			
Anaemia prevalence	1.2	0.68–2.0	1.1	0.65–2.0	0.6600	1.7	0.86–3.3	1.7	0.83–3.4	0.1470
Age group 6 to 11 years	0.92	0.63–1.4	1.1	0.71–1.6	0.7090	0.72	0.43–1.2	1.1	0.60–1.9	0.8620
Age group 12 to 17 years	1.4	0.86–2.3	1.7	0.98–2.8	0.0580	1.7	0.87–3.2	2.7	1.3–5.4	0.0060
Socioeconomic quintile 4	1.1	0.59–2.1	1.2	0.63–2.2	0.2415	0.91	0.34–2.5	1.1	0.39–2.9	0.0165
Socioeconomic quintile 3	1.4	0.69–2.7	1.4	0.70–2.8		1.2	0.41–3.4	1.3	0.45–3.8	
Socioeconomic quintile 2	1.3	0.65–2.4	1.2	0.64–2.3		1.5	0.55–4.0	1.5	0.57–4.2	
Socioeconomic quintile 1 (poorest)	2.3**	1.1–4.6	2.3	1.1–4.6		5.1***	1.8–14.2	5.1	1.8–14.7	

*Notes*: RRs in bold had univariable *P* < 0.2 and were entered in multivariable regression models; for correct interpretation of this table, if a variable was significant for moderate stunting but not severe stunting, it was still included, therefore on occasion moderate stunting adjusted RRs are significant when severe stunting adjusted RRs are not, and *vice versa. Ascaris* (categorical infection intensity), *N. americanus* (categorical infection intensity), *Ancylostoma* infection prevalence (binary), anaemia prevalence (binary), age group, sex and socioeconomic quintile were included in all multivariable regression models as exposure outcomes and covariates. A sex*N. americanus interaction is included in the model. Reference categories: no *Ascaris* infection, no *N. americanus* infection, female sex, no *N. americanus* infection in males (multivariable only), no *Ancylostoma* infection, no *Giardia* infection, no anaemia, age group 1–5 years, socioeconomic quintile 5 (wealthiest)

*Abbreviations*: *RR* relative risk, *ARR* adjusted relative risk, *CI* confidence interval; *P*, Wald test *P*-value

**P < 0.05, ***P < 0.01 in univariable analysis

^a^The *N. americanus* main effect, being *N. americanus* infection intensity relative to no *N. americanus* infection in females (because females are the reference group)

^b^The male sex main effect, being the relative risk of male sex relative to female sex when there is no *N. americanus* infection (reference group)

^c^ *N. americanus* infection intensity in males is relative to no *N. americanus* infection in males (because males and moderate- or heavy-intensity *N. americanus* infection are not the reference groups)

^d^Ten observations or less in subgroup; result should be interpreted cautiously

Normal growth (no stunting) is reference category, i.e. moderate and severe stunting need to be interpreted relative to this reference. *Ascaris* and *N. americanus* intensity infections defined according to following cut-points: *Ascaris*: heavy-intensity C_q_ ≤ 15.4, moderate-intensity C_q_ > 15.4 < 31, no infection C_q_ ≥ 31; *N. americanus*: heavy-intensity C_q_ ≤ 24.6, moderate-intensity C_q_ > 24.6 < 35, no infection C_q_ ≥ 35

Due to inclusion of a sex by *N. americanus* interaction term, we report results of the association between *N. americanus* and stunting separately for males and females. Females with *N. americanus* infection of any intensity had no significant association with stunting of any severity. Being male and having heavy-intensity *N. americanus* infection (relative to being male and having no *N. americanus* infection) was associated with significantly reduced risk of severe stunting. It is important to note, however, that the main effect of *N. americanus* infection intensity showed that there was no association with stunting in females (the reference category). The main effect for sex indicates that, in those with no *N. americanus* infection, being male was highly significantly associated with almost three times the risk of moderate stunting, and almost seven times the risk of severe stunting. Relative to children aged one to five, children aged 12 to 17 had almost twice the risk of moderate stunting, and almost three times the risk of severe stunting, although no significant associations were seen in children aged 6 to 11 years. Children in the poorest socioeconomic quintile had twice the risk of moderate stunting, and five times the risk of severe stunting, compared to children in the wealthiest socioeconomic quintile. Anaemia was not a risk factor for stunting.

### Factors associated with being underweight

The association between underweight and *N. americanus* infection is reported separately by age group because of the age group by *N. americanus* interaction term. In those aged one to five, *N. americanus* infection of any severity was not associated with being underweight. Whilst this same association was evident for children aged six to ten with *N. americanus* infection, the association with being severely underweight was only marginally non-significant for those having moderate *N. americanus* intensity of infection. Compared to children aged 1 to 5 years, being aged 6 to 10 years was significantly associated with three times the risk of being severely underweight, but no increased risk of being moderately underweight, in *N. americanus*-uninfected children. There was no association between intensity of *Ascaris* infection and being underweight in this population (Table 9). *Ancylostoma* infection was close to significance for being moderately underweight, but not for being severely underweight. The presence of *G. duodenalis* infection, or of anaemia, were also not significantly associated with being underweight. Being male was highly significantly associated with three times greater risk of being severely underweight compared to normal growth children, but this association was not evident for moderate levels of underweight. Household socioeconomic quintile was not associated with being underweight in these analyses, although the association with poorest socioeconomic quintile was only marginally not significant for being moderately underweight. With binary-coded underweight as outcome, a sensitivity analysis confirmed the consistency of results with the multinomial results.

**Table 9:** Relative risk ratios for being underweight, Manufahi District, Timor-Leste

	Moderate underweight (*n* = 253)					Severe underweight (*n* = 129)				
Parameter	RR	95% CI	ARR	95% CI	*P*	RR	95% CI	ARR	95% CI	*P*
*Ascaris* heavy-intensity	1.0	0.53–2.1	0.85	0.41–1.7	0.8925	1.6	0.72–3.5	1.2	0.49–2.8	0.9379
*Ascaris* moderate-intensity	1.1	0.60–2.1	1.0	0.52–1.9		1.5	0.73–3.3	1.0	0.46–2.3	
*N. americanus* heavy-intensity^a^	1.0	0.64–1.7	0.59	0.28–1.2	0.3584	0.85	0.47–1.5	0.66	0.25–1.8	0.1519
*N. americanus* moderate-intensity^a^	0.94	0.41–2.2	0.76	0.26–2.2		0.87^d^	0.31–2.5	0.12^d^	0.01–1.2	
Age group 6 to 10 years^b^	1.4	0.91–2.2	1.2	0.63–2.2	0.5930	2.8***	1.6–4.8	3.3	1.5–7.0	0.0030
Age group 6 to 10 years and *N. americanus* heavy-intensity infection^c^	1.4	0.80–2.5	1.8	0.67–4.8	0.2510	1.7	0.81–3.6	0.70	0.20–2.4	0.5740
Age group 6 to 10 years and *N. americanus* moderate-intensity infection^c^	1.8	0.39–8.2	2.1	0.30–13.9	0.4630	7.0	1.36–35.8	15.6	0.91–266.4	0.0580
*Ancylostoma* infection	2.5	0.88–6.8	2.8	0.94–8.1	0.0640	0.69^d^	0.15–3.2	0.86^d^	0.18–4.2	0.8480
*Giardia* infection	0.97	0.58–1.6				1.1	0.58–2.1			
Anaemia prevalence	0.99	0.56–1.8	0.98	0.54–1.8	0.9430	0.70	0.34–1.5	0.85	0.39–1.9	0.6820
Male sex	1.3	0.85–2.0	1.3	0.81–2.0	0.3040	2.4***	1.4–4.2	3.1	1.7–5.6	< 0.0001
Socioeconomic quintile 4	1.4	0.66–3.1	1.5	0.66–3.3	0.2606	0.57	0.20–1.6	0.66	0.23–1.9	0.3095
Socioeconomic quintile 3	1.1	0.46–2.4	1.1	0.48–2.6		1.0	0.36–2.8	1.1	0.37–3.0	
Socioeconomic quintile 2	1.1	0.53–2.5	1.2	0.54–2.7		0.87	0.33–2.3	0.96	0.36–2.6	
Socioeconomic quintile 1 (poorest)	2.4	1.0–5.6	2.5	1.0–6.1		2.0	0.72–5.8	2.2	0.76–6.6	

*Notes*: *Ascaris* (categorical infection intensity), *N. americanus* (categorical infection intensity), *Ancylostoma* infection prevalence (binary), anaemia prevalence (binary), age group, sex and socioeconomic quintile were included in all multivariable regression models as exposure outcomes and covariates. Being underweight not measured in children aged 11 to 17 years. An age group*N. americanus interaction is included in the model. Reference categories: No *Ascaris* infection, no *N. americanus* infection, age group 1–5 years, no *N. americanus* infection in age group 6 to 10 years (multivariable only), no *Ancylostoma* infection, no *Giardia* infection, no anaemia, female sex, socioeconomic quintile 5 (wealthiest)

*Abbreviations*: *RR* relative risk, *ARR* adjusted relative risk, *CI* confidence interval, *P*, Wald test *P*-value

**P < 0.05, ***P < 0.01 in univariable analysis

^a^The *N. americanus* main effect, being *N. americanus* infection intensity relative to no *N. americanus* infection in age group 1 to 5 years (reference age group)

^b^The age group 6 to 10 years main effect, being the relative risk of being aged 6 to 10 relative to being aged 1 to 5 when there is no *N. americanus* infection (reference group)

^c^ *N. americanus* infection intensity in age group 6 to 10 years is relative to no *N. americanus* infection in age group 6 to 10 years (because age group 6 to 10 years and moderate- or heavy-intensity *N. americanus* infection are not the reference groups)

^d^10 observations or less in subgroup; result should be interpreted cautiously

Normal weight is reference category, i.e. moderate and severe underweight need to be interpreted relative to this reference. *Ascaris* and *N. americanus* intensity infections defined according to following cut-points: *Ascaris*: heavy-intensity C_q_ ≤ 15.4, moderate-intensity C_q_ > 15.4 < 31, no infection C_q_ ≥ 31; *N. americanus*: heavy-intensity C_q_ ≤ 24.6, moderate-intensity C_q_ > 24.6 < 35, no infection C_q_ ≥ 35

### Factors associated with wasting

Although no intestinal parasites were associated with wasting in adjusted analyses, observation numbers in this model were very low for assessment of some categories (Table 10). Additionally, the presence of anaemia was not associated with wasting. Relative to being aged 1 to 5 years, being aged 6 to 11 years was associated with highly significant, threefold increased risk for being either moderately or severely wasted. Strikingly, this trend worsened amongst children aged 12 to 17 years, with four times the risk of moderate wasting, and seven times the risk of severe wasting, seen in these children relative to the youngest age group. There was no association between sex or socioeconomic status and categories of wasting. Given low numbers with this multinomial wasting outcome, a sensitivity analysis was conducted using wasting as a binary-coded outcome; results were consistent with the multinomial analyses, with age group the sole significant factor.

**Table 10:** Relative risk ratios for wasting, Manufahi District, Timor-Leste

	Moderate wasting (*n* = 145)					Severe wasting (*n* = 44)				
Parameter	RR	95% CI	ARR	95% CI	*P*	RR	95% CI	ARR	95% CI	*P*
*Ascaris* heavy-intensity	0.83	0.42–1.6	0.83	0.40–1.7	0.4465	0.51^a^	0.13–2.1	0.37^a^	0.07–1.9	0.4942
*Ascaris* moderate-intensity	0.73	0.38–1.4	0.64	0.32–1.3		1.1^a^	0.41–3.1	0.92^a^	0.32–2.7	
*N. americanus* heavy-intensity	1.7**	1.1–2.8	1.3	0.76–2.1	0.5964	2.1**	0.91–4.7	1.2	0.51–3.0	0.8431
*N. americanus* moderate-intensity	0.87^a^	0.35–2.1	0.91^a^	0.36–2.3		1.5^a^	0.40–5.6	1.4^a^	0.35–5.4	
*Giardia* infection	0.79	0.47–1.3				0.46^a^	0.17–1.2			
Anaemia prevalence	0.63	0.32–1.2	0.83	0.41–1.7	0.6140	0.66^a^	0.22–2.0	1.2^a^	0.37–3.8	0.7770
Male sex	1.3	0.84–1.9	1.2	0.79–1.9	0.3580	1.3	0.68–2.7	1.3	0.61–2.6	0.5340
Age group 6 to 11 years	3.0***	1.7–5.2	2.9	1.6–5.1	< 0.0001	3.5**	1.3–9.3	3.2	1.2–8.9	0.0220
Age group 12 to 17 years	4.9***	2.6–9.2	4.3	2.2–8.4	< 0.0001	7.8***	2.6–22.7	7.1	2.3–21.8	0.0010
Socioeconomic quintile 4	0.80	0.40–1.6	0.82	0.39–1.7	0.9181	0.89^a^	0.27–3.0	0.92^a^	0.27–3.2	0.8840
Socioeconomic quintile 3	0.72	0.35–1.5	0.77	0.35–1.7		1.3^a^	0.36–4.4	1.3^a^	0.35–4.6	
Socioeconomic quintile 2	0.69	0.34–1.4	0.79	0.37–1.7		0.60^a^	0.16–2.3	0.70^a^	0.18–2.7	
Socioeconomic quintile 1 (poorest)	0.66	0.31–1.4	0.68	0.30–1.5		0.59^a^	0.15–2.3	0.66^a^	0.16–2.7	

*Notes*: *Ascaris* (categorical infection intensity), *N. americanus* (categorical infection intensity), *Ancylostoma* infection prevalence (binary), anaemia prevalence (binary), age group, sex and socioeconomic quintile were included in all multivariable regression models as exposure outcomes and covariates. Insufficient *Ancylostoma* spp. observations to investigate in this regression model. No interactions were significant in this model. Reference categories: No *Ascaris* infection, no *N. americanus* infection, no *Ancylostoma* infection, no *Giardia* infection, no anaemia, female sex, age group 1–5 years, socioeconomic quintile 5 (wealthiest)

*Abbreviations*: *RR* relative risk adjusted for age^2^, *ARR* adjusted relative risk, *CI* confidence interval; *P*, Wald test *P*-value

**P < 0.05, ***P < 0.01 in univariable analysis

^a^Ten observations or less in subgroup; result should be interpreted cautiously

Normal growth (no wasting) is reference category, i.e. moderate and severe wasting need to be interpreted relative to this reference. *Ascaris* and *N. americanus* intensity infections defined according to following cut-points: *Ascaris*: heavy-intensity C_q_ ≤ 15.4, moderate-intensity C_q_ > 15.4 < 31, no infection C_q_ ≥ 31; *N. americanus*: heavy-intensity C_q_ ≤ 24.6, moderate-intensity C_q_ > 24.6 < 35, no infection C_q_ ≥ 35

## Discussion

In this first investigation of STH associations with haemoglobin and child development indices in Manufahi District, Timor-Leste, a generally lower prevalence of anaemia than results reported previously [17] was observed, with 11% prevalence in children aged 1– < 5 years, and 18% prevalence in reproductive-aged women (18– < 45 years of age). For women, anaemia whilst moderately lower than reported previously [17], was significantly more frequent than in males of the same age, confirming the serious disease burden in this population group. This likely reflects the well-reported impact of pregnancies and menstruation on iron stores [34]. Anaemia in mothers is itself a risk factor for stunting or wasting in offspring. Iron-folic acid supplementation for pregnant women has been implemented by the Ministry of Health across all districts of Timor-Leste since 2003, with 61% of pregnant women reporting taking supplements in 2009–2010 [16].

Despite the difference in prevalence, being female was not demonstrated to be a risk factor for anaemia in adults or children. Interestingly, neither *N. americanus* nor *Ascaris* of either class of infection intensity were significant risk factors, this despite the well-recognised association between hookworm infection and blood loss. Whilst *N. americanus* is implicated in blood loss, it causes measurably less blood loss than *Ancylostoma duodenale* [10]. Blood loss due to parasite infection needs to be greater than nutritional reserves and required intake for anaemia to develop [35]. In this population the prevalence of the more pathogenic hookworm species *Ancylostoma duodenale* was very low, representing a possible explanation for the weak association between STH (particularly hookworm) and anaemia. Similar negligible associations have been identified in *N. americanus*-endemic populations elsewhere [35]. Socioeconomic status and age were not important risk factors for anaemia in adults, but were important, highly significant, risk factors for children. Different risk factor associations between children and adults point to the need to conduct further age-stratified analyses, ideally with children aged less than five analysed separately due to the higher prevalence of anaemia in this age group; observation numbers limited further age-stratification in our analysis. Anaemia can be caused by multiple concurrent factors including inadequate dietary iron, and it is inherently difficult to control for all of these in epidemiological studies. The lack of other identified risk factors in our models would suggest that additional unmeasured factors may be influencing these results. Of note, the prevalence of malaria had dramatically declined in Timor-Leste prior to the commencement of this study [36], indicating that this is not a likely confounding factor.

In the study area, an extremely high prevalence of stunting, underweight and wasting in children were observed compared to the international reference population, with considerable proportions of severe stunting, underweight and wasting. These are higher than national estimates, possibly reflecting the rurality of the study communities. Our reported prevalence, whilst being at a district, not national level (and therefore perhaps more susceptible to small geographic area fluctuations), are amongst some of the highest reported rates in the world [37]. This is despite relatively low community prevalence of anaemia. Proportions of child wasting in particular are well above the 15% level of severity classified as critical [38]. Wasting represents rapid and severe malnutrition such as starvation, although it can also be the result of chronic unfavourable conditions [38]. With strong links between wasting and child mortality [38] this level is considered to be a public health emergency [39] that requires immediate response. However, as a cautionary note, the application of the 2006 WHO international reference standards to the Timorese population has not been assessed, and there is a possibility of this population being of a smaller stature than the international standards, leading to overstated morbidity. Further investigations into applicability of these thresholds within Timor-Leste are required.

Stunting is often associated with poor nutrient availability *in utero* and the neonatal period from maternal breast milk, exacerbated by continuing poor nutrient supply during the period of introduction of solids [40]. It represents a period of chronic malnutrition during the most rapid growth period of life, leading to long-term and often permanent failure to attain linear growth. Wasting and stunting share direct and underlying causal factors, but it is not yet well understood how much wasting contributes to stunting and *vice versa* [41]. There are ongoing nutritional initiatives in Manufahi District including provision of food at schools. However, given the prevalence of wasting, stunting and underweight, there is an urgent need to further investigate nutrition in this community and enhance strategies to ensure that children are receiving adequate nutrition. The greater ARRs for severe stunting and wasting in older compared to younger children may reflect more food security for young children following the end of Indonesian occupation of Timor-Leste.

After controlling for socioeconomic status, sex, anaemia prevalence and age, there were few STH associations with child development indices, and most of the associations found fell short of the 5% significance threshold. Of importance were the sex by *N. americanus* interaction for stunting, and the age group by *N. americanus* interaction for underweight: the interaction terms highlight complex interrelationships occurring in the population, and main effects of *N. americanus* intensity of infection in females were not significantly associated with morbidity. It should not be interpreted from this analysis that *N. americanus* infection is associated with reduced risk of either stunting or being underweight. There was no other indication of association between either *N. americanus* or *Ascaris* spp. infection intensities with stunting, underweight or wasting outcomes. The other significant helminth-associated finding was for an association of *Ancylostoma* infection with moderate and severe stunting, although trends were not in the expected direction, with *Ancylostoma* infection associated with reduced risk of severe stunting (Table 6). The prevalence of *Ancylostoma* in this population was low, at 4.7%, leading to low numbers in regression models, and inability to categorise *Ancylostoma* into classes of infection intensity. Further, it was due to observed differences in the relationship between sex, age, *N. americanus* infection intensity, and stunting, that interactions were investigated. It could be that this underlying complex relationship also affected *Ancylostoma*. This finding of a ‘protective’ effect therefore needs to be interpreted cautiously.

The most significant risk factors for stunting and underweight were, generally, being male, and older child age groups, with additionally for stunting, being in the poorest socioeconomic quintile. Anaemia did not emerge as a risk factor for either stunting or underweight in this population. Underweight as a measure of child development is more variable than stunting, being influenced by both height and weight [38]. This may explain why poverty emerged as a risk factor for stunting but not being underweight in this population. Poverty could be a major contributing factor to chronic nutrient deprivation at critical rapid growth stages in this community. High stunting rates are usually indicative of poor socioeconomic conditions [38]; these rates may represent a legacy of conflict in this country. No risk factors were found for wasting with the exception of a greatly increased risk with older child age group for both moderate and severe wasting. These results, coupled with high STH prevalence, point to unmeasured risk factors in our study population, likely including nutritional risk factors, but also possibly genetic, behavioural and environmental risk factors.

Given high STH prevalence, the lack of other STH associations with morbidity in our models is surprising. However, this could be linked to the low level of anaemia and its negligible associations with child development outcomes in this population, given the most likely causal pathway between STH and morbidity is the role of hookworms contributing to blood loss and anaemia, with impacts on child development being more indirect. STH-morbidity associations have not been consistently found in studies of many different designs, contributing to a picture of complexity in understanding STH impacts on morbidity in populations that are often suffering multiple potentially interacting insults associated with poverty and deprivation (reviewed in [11]).

An alternative reason for the lack of STH associations with morbidity in our analysis may be that *N. americanus* morbidity may have been inadvertently overemphasised in studies that do not differentiate the hookworm genera (e.g. *Necator* and *Ancylostoma*). It could be that the burden of hookworm stems predominantly from *Ancylostoma* infection, and not *N. americanus*. This has been raised previously [10, 42]. Further epidemiological studies investigating differential morbidity, using advanced diagnostic methods, are required.

Studies reporting morbidity associations with STH show varied results (reviewed in [11]). The lack of consistent associations between STH and morbidity outcomes, particularly child-related *z*-scores, is problematic for quantifying disease burden. Early estimates of the global burden of disease from STH had widely varying ranges, primarily based on the interpretation of morbidity in school-aged children [43]. Of particular importance is that, historically, it was the perceived importance of disease in school-aged children that became a crucial factor in international advocacy and development of mass school-based STH control programmes [43]. However a Cochrane systematic review has concluded that there is “substantial evidence that deworming does not improve average nutritional status or haemoglobin” [44], although this conclusion is disputed due to there being insufficient evidence in existence to confirm or refute the findings, despite research efforts [11]. Lack of evidence likely reflects the underlying heterogeneity of STH in different populations, alongside numerous potentially influencing factors such as community nutrition, poverty and comorbidities. This highlights the importance of continuing detailed investigations into morbidity associations, in an attempt to meet evidence shortfalls. New technologies such as qPCR will potentially play a very important role in generating this evidence.

Historically, it has been heavy-intensity of infection that has been considered by the WHO as contributing to greatest morbidity within the population, therefore in this analysis the decision was made to categorise between heavy- and moderate- rather than moderate- and light-intensity infections, so as to compare the heavy-morbidity category in the epidemiological analyses. Possibly, if the moderate and heavy-intensity infection categories were combined, to demonstrate a category of light-intensity infection, it could have affected the results in a population that was showing associations with heavy-intensity infection and morbidity, by ‘hiding’ covariate relationships. However, as no strong associations between heavy-intensity STH and morbidity emerged, in this population the results may not have been affected substantially with a different categorisation of the intensity variable.

Population-based studies [27, 30–32, 45] are increasingly using PCR as a diagnostic tool for STH. However, using infection intensity has not been adequately undertaken to date. Of C_q_ cut-points that have been assigned, only one prior investigation was found that specified how cut-points were allocated; this study also used dilution experiments from gene fragments for each species [30]. PCR data have therefore not yet been sufficiently validated using a wide range of infection intensities across populations, by contrast to categories of epg which are widely used for microscopy-derived measures of infection intensity despite their not very well acknowledged methodological weaknesses [42]. Based on Kato-Katz alone, our study population could be considered to have mainly ‘light intensity’ infections. This highlights an extremely important aspect of current STH diagnostics. Semi-quantitative PCR is considered to be more sensitive than Kato-Katz and thus traditional Kato-Katz-based classifications likely underestimate intensity of infection. For qPCR, sensitivity can be high enough to detect DNA from a single egg or L1 larva. Specificity of PCR-based tests is very high; offering a higher level of species-specificity. For example, *Ancylostoma caninum* (dog hookworm) has recently been detected in a significant proportion of human stool specimens collected in Tamil Nadu, India [46]. Using qPCR diagnostic tests, the definition of light-intensity infections from epg could now be called into question. In the absence of diagnostic ‘gold standards’ [29], and the increasing use of copro-diagnostic technologies, validating infection intensity using qPCR measures will become an increasingly important requirement. This analysis provides the first assignment of C_q_ cut-points based on an algorithm correlating infection intensity as measured by C_q_ value to an epg equivalent using the same faecal specimens. Major statistical inaccuracies can arise from setting data-driven cut-points [47], and this analysis should be seen as exploratory. Ideally further investigations of C_q_ -epg correlations are required. Further, population-based, and/or mathematical modelling studies are required to derive C_q_ cut-points that can be applied across populations.

### Limitations and strengths

A limitation of this analysis is that there were insufficient observations to investigate anaemia risk factors in children under five, which was highly desired, or age-related differences for child development indices. Power calculations also indicated that there was power to detect risk factors for anaemia of odds ratios above 3.3, and for child morbidity relative risks of generally above 1.4. The prevalence was generally higher in young children; these are the most formative and highest-velocity growth years. Future research should investigate differential associations by age.

A further limitation of the study is that it was not possible to formally adjust for malaria prevalence. However, one of the largest recorded reductions in malaria incidence has recently been reported for Timor-Leste, with a 97% decrease in reported malaria incidence over 2006–2012 [36] to a very low level. This provides evidence for a low underlying incidence of malaria in these communities and hence this is believed to be a minor limitation. A final limitation of this study is, as it was a parasitological investigation, food intake assessment was not considered in the design. Important additional information could be provided by such an assessment in this population.

A major strength of this study is that it provides the first quantitative assessment of the role of STH on measures of morbidity in Timor-Leste, using advanced parasitological and epidemiological methods. This provides an important epidemiological evidence base to inform policy and programmatic planning. In particular, differentiation of *N. americanus* and *Ancylostoma* coupled with the low morbidity association overall, has enabled us to hypothesise that *N. americanus* morbidity effects have possibly been overstated in settings where less sensitive STH diagnostic tools have been used. This is a very important research priority to investigate further. Additionally, in conducting a parasitological survey, the need to investigate nutritional epidemiology in this district has been identified. Such investigations should commence as a priority.

## Conclusions

This report provides the first assessment of STH associations with haemoglobin, stunting, wasting and underweight anthropometric measures in Timor-Leste. In this high-prevalence setting, only weak associations between STH of any species and developmental measures were found. Despite this, high prevalence of stunting, underweight and wasting in children illustrates the urgent need for investigating food quality and quantity in this community and providing nutritional enhancement, for example *via* micro- and macro-nutrient supplements. Additionally, regardless of the lack of association found between intensity of STH infection and morbidity in this study population, the high prevalence of STH provides a strong justification for introducing integrated STH control strategies. Deworming will reduce STH infections, but further nutritional interventions are going to be required to improve health.

## Additional file

Additional file 1: Text 1. Investigation of qPCR data and subsequent categorisation into classes of *Necator americanus* and *Ascaris* spp. infection intensity. **Figure S1.** *Necator americanus* and *Ascaris* spp. intensity of infection (C_q_-value) distributions. Uninfected people were excluded from these figures for purposes of scale. **Text 2.** Use of receiver-operating characteristic curves to statistically assign intensity of infection cut-points. **Text 3.** Use of epg-C_q_ algorithm to statistically assign intensity of infection cut-points. **Table S1** *Ascaris* spp. and *Necator americanus* intensity of infection quantification cycle (C_q_) cut-points between heavy and moderate morbidity assigned from receiver-operating characteristic curves. (DOCX 17 kb)

## Abbreviations

AOR: Adjusted odds ratio
ARR: Adjusted relative risk
AUC: Area under ROC curve
BMI: Body mass index
BMIZ: BMI-for-age
CI: Confidence interval
C_q_: Quantification cycle
DNA: Deoxyribonucleic acid
Epg: Eggs per gram of faeces
HAZ: Height-for-age
OR: Odds ratio
qPCR: Quantitative real-time polymerase chain reaction
RCT: Randomised controlled trial
ROC: Receiver-operating characteristic curve
RR: Relative risk
SD: Standard deviation
STH: Soil-transmitted helminth
WASH: Water, sanitation and hygiene
WAZ: Weight-for-age
WHO: World Health Organization
