# Supplementary material for: Investigations into the association between soil-transmitted helminth infections, haemoglobin and child development indices in Manufahi District, Timor-Leste
Source: Parasit Vectors. 2017 Apr 19;10:192. doi: 10.1186/s13071-017-2084-x (PMC5395746; doi:10.1186/s13071-017-2084-x)
Supplement: Additional file 1: Text 1. — Investigation of qPCR data and subsequent categorisation into classes of Necator americanus and Ascaris spp. infection intensity. Figure S1. Necator americanus and Ascaris spp. intensity of infection (Cq-value) distributions. Uninfected people were excluded from these figures for purposes of scale. Text 2. Use of receiver-operating characteristic curves to statistically assign intensity of infection cut-points. Text 3. Use of epg-Cq algorithm to statistically assign intensity of infection cut-points. Table S1 Ascaris spp. and Necator americanus intensity of infection quantification cycle (Cq) cut-points between heavy and moderate morbidity assigned from receiver-operating characteristic curves. (DOCX 17 kb) [file 13071_2017_2084_MOESM1_ESM.docx]

**Additional file 1. Text 1.** Investigation of qPCR data and subsequent categorisation into classes of *Necator americanus* and *Ascaris* spp. infection intensity

In this study, C_q_ values were distinctly bimodal, particularly for *Ascaris* spp. (Figure S1). Statistically, bimodal explanatory variables present challenges for interpretation of model coefficients, even if the residuals are Normally distributed, as there is known poor fit to linear, quadratic or cubic equations [1]. Bimodality in explanatory variables is often ignored. However, it was important to investigate in our samples because cut-points were being assigned to generate categories of intensity of infection. The distribution of C_q_ was examined by sex, age group, socioeconomic quintile, and STH co-infection, to investigate whether the modes varied with these variables. However, the distribution, and modes, appeared consistent across all groups. For ease of interpretation and for comparison with other studies, untransformed C_q_ values were categorised in these analyses.


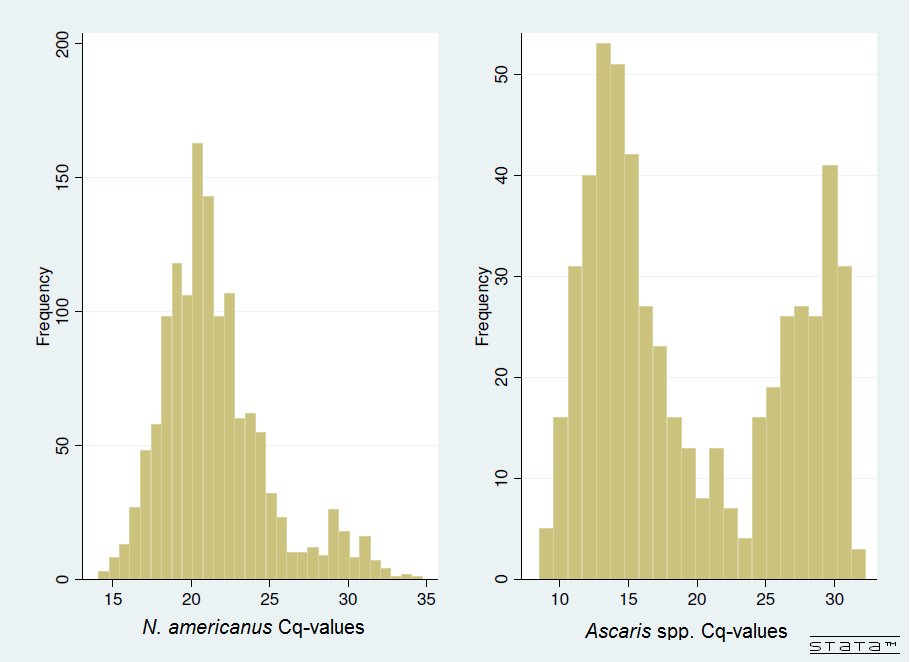


**Additional file 1: Figure S1** *Necator americanus* and *Ascaris* spp. intensity of infection (C_q_-value) distributions. Uninfected people were excluded from these figures for purposes of scale

**Additional file 1. Text 2.** Use of receiver-operating characteristic curves to statistically assign intensity of infection cut-points

Receiver-operating characteristic (ROC) curves show the trade-off between sensitivity and specificity, using the area under the ROC curve (AUC). Sensitivity is the proportion of individuals with the outcome of interest who are correctly classified according to the C_q_ categorisation. Specificity is the proportion of individuals without the outcome who are correctly classified, where presence or absence of the outcome is defined by a “gold standard” which is assumed to correctly classified individuals. An AUC of 0.5-0.7 is interpreted as having poor predictive accuracy; 0.7-0.9 reasonable predictive accuracy; and >0.9 very good predictive accuracy [2].

A morbidity score was developed based on the presence/absence of moderate and severe stunting, wasting and underweight in children aged 1<10 years, with the following assignment: moderate stunting = 1, moderate wasting = 1, moderate underweight = 1, severe stunting = 2, severe wasting = 2, severe underweight = 2. These values were summed to a maximum possible score per child of 6. These scores were developed for the subgroup of children who were at the correct age to have all measures (i.e. children aged 1<10 years), and for purposes of generating scores we assumed that the relationship with C_q_ was the same for each of the morbidity measures. The morbidity score was then categorised as two versions of a binary variable (scores 0-1, scores 2-3, scores 4-6; whereby the cut-point between 1 and 2 represented the cut-point between low and moderate morbidity, and the cut-point between 3 and 4 represented the cut-point between moderate and high morbidity) for running ROCs.

ROC analyses initially compared severe versus non-severe morbidity, and then moderate versus low morbidity. These were calculated as nonparametric curves, whereby the points on the curve were generated by using each possible outcome of the C_q_ as a classification cut-point and computing the corresponding sensitivity and specificity. For each of the intensity of infection outcomes, the C_q_ value associated with the least distance between the ROC curve and ‘perfect’ classification was selected (i.e. the point on the ROC curve that had the greatest optimisation with sensitivity and specificity). Due to poor predictive capacity (Table S1), the assignment of cut-points between classes of infection intensity was discontinued. This demonstrated the weak underlying relationship between STH and morbidity in this population, with no apparent signal. That is, the test measure had no discriminating ability and an outcome could just as accurately have been based on chance. ROC analysis is an important statistical tool that can be used to assign cut-points, however it relies on the existence of a relationship between predictor and outcome.

**Additional file 1. Text 3.** Use of epg-C_q_ algorithm to statistically assign intensity of infection cut-points

An algorithm to assign intensity of infection based on approximations of epg was generated from seeding experiments, and was based on the linear relationship between the log_10_ of epg and C_q_ value [3]. Briefly, hookworms were freshly isolated from *N. americanus* infected stool samples and *Ascaris suum* eggs were purchased from Excelsior Sentinel Inc. (Ithaca, NY) and stored in 5% potassium dichromate. *Ascaris* and hookworm eggs were purified separately [4], and the concentration of eggs in each sample determined by microscopy. Purified eggs were diluted to produce a range of concentrations of eggs; *Ascaris* eggs were prepared in triplicate in concentrations ranging from 200,000 epg to 5 epg and hookworm eggs were prepared in duplicate with concentrations ranging from 6,000 epg to 250 epg. Both *Ascaris* and hookworm eggs at each of the concentrations were subject to DNA extraction and multiplex PCR. These standard curves were used for the interpolation of the relationship between the log-transformed epg and log-transformed PCR intensity to determine a relationship between C_q_values and epg. For *N. americanus* the equation was epg=10^(-0.43Ct + 14.88)^, and for *Ascaris*, epg=10^(-0.275Ct + 9.622)^ [3].

The rationale for applying the recovery factor of 0.2 was based on a 20% recovery rate determined for faecal flotation of *Ascaris* eggs (R. Traub, unpublished data). Specifically, this experiment demonstrated average recovery of *A. suum* eggs spiked into parasite negative faeces provided a recovery rate of 20% on standard faecal flotation using sodium nitrate. Poor recovery of similar geohelminth parasites (ascarids, trichurids and strongyles) in the faeces of animals using sodium nitration flotation has been proven in the past. For example, O’Grady and Slocombe [5] found 50% of eggs trapped in the faeces or retained in the strainer and 16-29% of eggs retained in the flotation solution. Additionally, average recovery of *A. lumbricoides* eggs from soil (clay, sand and loam) using sodium nitration flotation ranged from 2.2 to 15% [6]. Further, despite single faecal flotation being more sensitive in detecting hookworm eggs in human faeces compared with quadruple Kato-Katz, the egg counts are comparable [7]. Standard curves obtained from well-defined spiked controls of *N. americanus* and *Ascaris* eggs in negative faeces allowed the determined C_q_ values (converted to infection intensity) to be determined [3]. This robust experiment supports aforementioned data that egg counts as determined by flotation and Kato-Katz are gross underestimates of 'true' egg counts in faeces. Unfortunately experiments to reclassify infection intensity levels using next-generation diagnostic approaches have not been attempted.

**Additional file 1. Table S1** *Ascaris* spp. and *Necator americanus* intensity of infection quantification cycle (C_q_) cut-points between heavy and moderate morbidity assigned from receiver-operating characteristic curves

| **STH** | **C_q_ cut-point** | **Distance** | **AUC score** |
| --- | --- | --- | --- |
| *N. americanus* | C_q_23 | 0.70 | 0.48 |
| *Ascaris* spp. | C_q_17 | 0.66 | 0.50 |

*Abbreviations:* C_q_, quantification cycle; ROC, receiver operating characteristic curve; AUC, area under the ROC.

*Notes:* distance is calculated as d=√[(1-sensitivity)^2^ + (1-specificity)^2^], with Ct cut-point selection based on the point on the ROC curve with least distance to the point of ‘perfect’ classification.

**Additional File References**

1. Royston P, Ambler G, Sauerbrei W. The use of fractional polynomials to model continuous risk variables in epidemiology. Int J Epidemiol. 1999;28(5):964-74.
2. Brooker S, Hay SI, Bundy DA. Tools from ecology: useful for evaluating infection risk models? Trends Parasitol. 2002;18(2):70-4.
3. Llewellyn S, Inpankaew T, Nery S, Gray DJ, Verweij JJ, Clements ACA, et al. Application of a multiplex quantitative PCR method for assessing intestinal parasite infection in a prospective controlled interventional study in Timor-Leste. PLoS Negl Trop Dis. 2016:Jan 28;10(1):e0004380.
4. Kotze AC, Coleman GT, Mai A, McCarthy JS. Field evaluation of anthelmintic drug sensitivity using in vitro egg hatch and larval motility assays with *Necator americanus* recovered from human clinical isolates. Int J Parasitol 2005;35:445-53.
5. O'Grady MR, Slocombe JO. An investigation of variables in a fecal flotation technique. Can J Comp Med. 1980;44(2):148-57.
6. Ajala MO & Asaolu SO. Efficiency of the salt flotation technique in the recovery of *Ascaris lumbricoides* eggs from the soil. J Helminthol. 1995;69(1):1-5.
7. Inpankaew T, Schȁr F, Khieu V, Muth S, Dalsgaard A, Marti H, et al. Simple fecal flotation is a superior alternative to quadruple Kato Katz smear examination for the detection of hookworm eggs in human stool. PLoS Negl Trop Dis 2014;8(12): e3313.
